# Host carbon sources modulate cell wall architecture, drug resistance and virulence in a fungal pathogen

**DOI:** 10.1111/j.1462-5822.2012.01813.x

**Published:** 2012-06-05

**Authors:** Iuliana V Ene, Ashok K Adya, Silvia Wehmeier, Alexandra C Brand, Donna M MacCallum, Neil A R Gow, Alistair J P Brown

**Affiliations:** 1 Aberdeen Fungal Group, School of Medical Sciences, Institute of Medical Sciences, University of AberdeenForesterhill, Aberdeen, AB25 2ZD, UK; 2 Division of Biotechnology and Forensic Sciences, School of Contemporary Sciences, University of Abertay DundeeDundee, DD1 1HG, UK

## Abstract

The survival of all microbes depends upon their ability to respond to environmental challenges. To establish infection, pathogens such as *Candida albicans* must mount effective stress responses to counter host defences while adapting to dynamic changes in nutrient status within host niches. Studies of *C. albicans* stress adaptation have generally been performed on glucose-grown cells, leaving the effects of alternative carbon sources upon stress resistance largely unexplored. We have shown that growth on alternative carbon sources, such as lactate, strongly influence the resistance of *C. albicans* to antifungal drugs, osmotic and cell wall stresses. Similar trends were observed in clinical isolates and other pathogenic *Candida* species. The increased stress resistance of *C. albicans* was not dependent on key stress (Hog1) and cell integrity (Mkc1) signalling pathways. Instead, increased stress resistance was promoted by major changes in the architecture and biophysical properties of the cell wall. Glucose- and lactate-grown cells displayed significant differences in cell wall mass, ultrastructure, elasticity and adhesion. Changes in carbon source also altered the virulence of *C. albicans* in models of systemic candidiasis and vaginitis, confirming the importance of alternative carbon sources within host niches during *C. albicans* infections.

## Introduction

Most microbes inhabit microenvironments that are in a constant state of flux. This is particularly true for clinically important microbial pathogens which must counter the biochemical and immunological insults imposed by their host. Pathogens must activate appropriate responses to these local environmental stresses while adapting to and assimilating the available nutrients within that niche.

*Candida albicans* is a major fungal pathogen of humans that can cause a variety of infections (Calderone and Clancy, 2011). *C. albicans* is carried as a relatively harmless commensal in the oral, gastrointestinal and urogenital microflora of 40–80% of the population. Mucosal infections (thrush) can arise when immunological defects or pharmacological interventions affect host–fungus interactions, and a significant proportion of these infections can be recurrent (Calderone, 2002). In severely immunocompromised patients *C. albicans* can thrive in the bloodstream, ultimately colonizing and forming lesions on internal organs (Perlroth *et al*., 2007; Pfaller and Diekema, 2010). Up to half of these systemic infections are fatal (Perlroth *et al*., 2007). Therefore, *C. albicans* can thrive within diverse niches in its human host.

Clearly to cause infection, *C. albicans* must grow and divide in these diverse niches. Cells must assimilate locally available carbon sources which can include fermentable sugars and non-fermentable carbon sources (Lorenz and Fink, 2001; Lorenz *et al*., 2004; Piekarska *et al*., 2006; Vieira *et al*., 2010; Ueno *et al*., 2011). Physiologically relevant sugars include glucose, fructose and galactose. However, these sugars are only present at low levels and are even absent in many host niches. Consequently, other non-fermentable carbon sources become essential to support yeast growth and metabolism *in vivo* (Piekarska *et al*., 2006; Vieira *et al*., 2010; Ueno *et al*., 2011). These alternative carbon sources include amino acids and organic acids. For example, lactic acid is present in ingested foods, produced via host metabolic activity, generated by endogenous lactic acid bacteria in the urogenital and gastrointestinal tracts, and is an important carbon source for *Candida* in the intestine (Ueno *et al*., 2011).

Thinking about carbon source utilization in yeasts is strongly influenced by the *Saccharomyces cerevisiae* paradigm. When this relatively benign model yeast is faced with a mixture of carbon sources it preferentially utilizes sugars such as glucose before assimilating alternative carbon sources (Johnston, 1999). This hierarchical metabolic activity is regulated by a complex glucose signalling network involving the co-ordinated action of AMP kinase, protein kinase A and sugar receptor repressor signalling pathways (Kim and Johnston, 2006). This signalling network downregulates metabolic pathways required for the assimilation of alternative carbon sources at both transcriptional and post-transcriptional levels (Yin *et al*., 1996; Sexton *et al*., 2007; Gancedo, 2008; Hedbacker and Carlson, 2008; Zaman *et al*., 2008; Askew *et al*., 2009). To some extent this signalling network and the downstream regulatory mechanisms have been conserved in *C. albicans* (Sabina and Brown, 2009). However, recent work has shown that there has been major transcriptional rewiring as well as significant divergence in the components that regulate carbohydrate and lipid metabolism in *C. albicans* (Martchenko *et al*., [Bibr b42]; Lavoie *et al*., 2009). Indeed, unlike *S. cerevisiae*, *C. albicans* is classified as a glucose-negative yeast because it continues to respire in the presence of glucose (Niimi *et al*., 1988). Clearly, benign and pathogenic yeasts have evolved to respond differentially to glucose, reflecting their contrasting niches. *S. cerevisiae* is thought to have evolved under conditions of ‘*feast and famine*’, rapidly exploiting fermentable sugars before switching to alternative carbon sources (Johnston, 1999). In contrast, *C. albicans* often inhabits niches that are glucose-limited but rich in alternative carbon sources. The physiological robustness of this pathogen *in vivo* is probably enhanced by its ability to assimilate multiple carbon sources simultaneously, rather than the sequential use of ‘preferred’ and then ‘non-preferred’ carbon sources (Brown *et al*., 2007).

Genome-wide expression profiling studies have confirmed the ability of *C. albicans* to undergo rapid metabolic transitions (Lorenz *et al*., 2004; Rodaki *et al*., 2009). For several fungi, as well as some bacteria (e.g. *Mycobacterium tuberculosis*), the glyoxylate cycle plays an important role in pathogenesis and is required for full virulence in the host (Lorenz and Fink, 2001). Recent work has reinforced the essentiality of carbon metabolism for fungal pathogenicity (Price *et al*., 2011; Vylkova *et al*., 2011). In particular, the induction of lactate transporters and metabolic enzymes upon macrophage internalization (Lorenz *et al*., 2004) suggests that high concentrations of lactate are present in the phagosome. As peroxisomal fatty acid β-oxidation is not essential for virulence (Piekarska *et al*., 2006), it is conceivable that lactate assimilation and utilization might support flux through the glyoxylate cycle thereby contributing to the survival of the pathogen under glucose-limiting conditions. The dependence of *Candida glabrata* upon lactate assimilation in certain host niches further strengthens this view (Ueno *et al*., 2011). However, most virulence studies have been performed using cells grown in rich glucose-containing media, and thus the impact of carbon source on infection development has yet to be addressed.

How does growth on different carbon sources affect the ability of yeasts to adapt to dynamic changes in their environment? In *S. cerevisiae*, glucose downregulates the core stress response via cAMP signalling, rendering cells more sensitive to environmental insults. Following the diauxic shift to alternative carbon sources, this yeast becomes more stress-resistant (Gounalaki and Thireos, 1994; Stanhill *et al*., 1999; Görner *et al*., 2002). The situation is less clear in *C. albicans* as most studies of stress adaptation have been performed on glucose-grown cells. The core stress response has diverged significantly in *C. albicans* (Enjalbert *et al*., 2003; 2006; Ramsdale *et al*., 2008), and while glucose exposure and cAMP signalling do influence stress resistance in *C. albicans* (Wilson *et al*., 2007; Rodaki *et al*., 2009), this is by unknown mechanisms. The effects of alternative carbon sources upon stress resistance of *C. albicans* remain unexplored despite the fact that this yeast depends upon such nutrients in many host niches (Lorenz and Fink, 2001; Barelle *et al*., 2006).

Similarly, the impact of growth on alternative carbon sources on the *C. albicans* cell wall remains largely unexplored, although the cell wall is a key effector of cell morphology and robustness, the major point of contact with the host, an active modulator of host immune defences and a prime target for antifungal drugs. Furthermore, a large proportion of assimilated carbon is invested in cell wall biogenesis as the cell wall comprises ∼ 30% of the yeast cell dry weight. The *C. albicans* yeast cell wall contains three main components: mannoproteins (∼ 39%), β-glucans (∼ 59%) and chitin (∼ 2%) (Aguilar-Uscanga and François, 2003). At a gross level, the composition of the *S. cerevisiae* cell wall has been shown to vary considerably in response to changes in carbon source, temperature, pH and aeration (Aguilar-Uscanga and François, 2003). Several observations imply mutual interactions between stress adaptation and cell wall architecture in *C. albicans*. Mannan chain length and complexity is altered when cells are grown in blood or serum rather than standard laboratory growth media (Kruppa *et al*., 2011). The stress-activated protein kinase Hog1 (Smith *et al*., 2004) influences cell wall biosynthesis (Eisman *et al*., 2006), partly by regulating chitin synthesis (Munro *et al*., 2007; Walker *et al*., 2008). Also, in response to certain stresses, the cell integrity pathway activates cell wall biogenesis (Navarro-Garcia *et al*., 1998; Eisman *et al*., 2006; Munro *et al*., 2007; Blankenship *et al*., 2010). In addition, stressors have recently been shown to influence mannan structures in *C. albicans* (Koyama *et al*., 2009). However, few studies have examined the influence of growth conditions on the cell wall (Kulkarni *et al*., 1980; Aguilar-Uscanga and François, 2003; Kawahata *et al*., 2006; Kruppa *et al*., 2011), and none has examined this in any detail despite its relevance to disease progression.

In this study we first characterized the impact of carbon source on the *C. albicans* cell wall, because of the critical role of the fungal cell wall during infection. We compared cells grown on glucose versus the physiologically relevant carboxylic acid, lactate, with a view to comparing this gluconeogenic carbon source with the carbon source classically used for experimental dissection of stress adaptation in *C. albicans*. We chose sodium lactate for these detailed cell wall analyses because lactate has been used previously to study metabolic flexibility (Vieira *et al*., 2010) and stress resistance (Rodaki *et al*., 2009). Lactate is an abundant carbon source in the intestine and vaginal mucosa and is produced at high rates by red blood cells, brain and muscle (Buchalter *et al*., 1989; Ueno *et al*., 2011). It is also found clinically in isotonic solutions commonly used after trauma, surgery or burn injury (lactated Ringer's solutions, Hartmann’ solution), all of which increase the risk of systemic candidiasis (Pfaller and Diekema, 2010). Having demonstrated the impact of lactate, we then confirmed that *C. albicans* is affected by other carbon sources that are commonly found in the host, including galactose, fructose, oleic acid, pyruvate, sorbitol and amino acids.

Our analyses revealed the major effects of carbon source on the architecture as well as the biochemical and biophysical properties of the cell wall. Alternative carbon sources strongly influenced adhesion, stress adaptation and drug resistance in *C. albicans in vitro*, as well as the virulence of this major pathogen in *in vivo* models of systemic candidiasis and vaginitis.

## Results

### Carbon source affects cell wall architecture

**Fig. 1:**
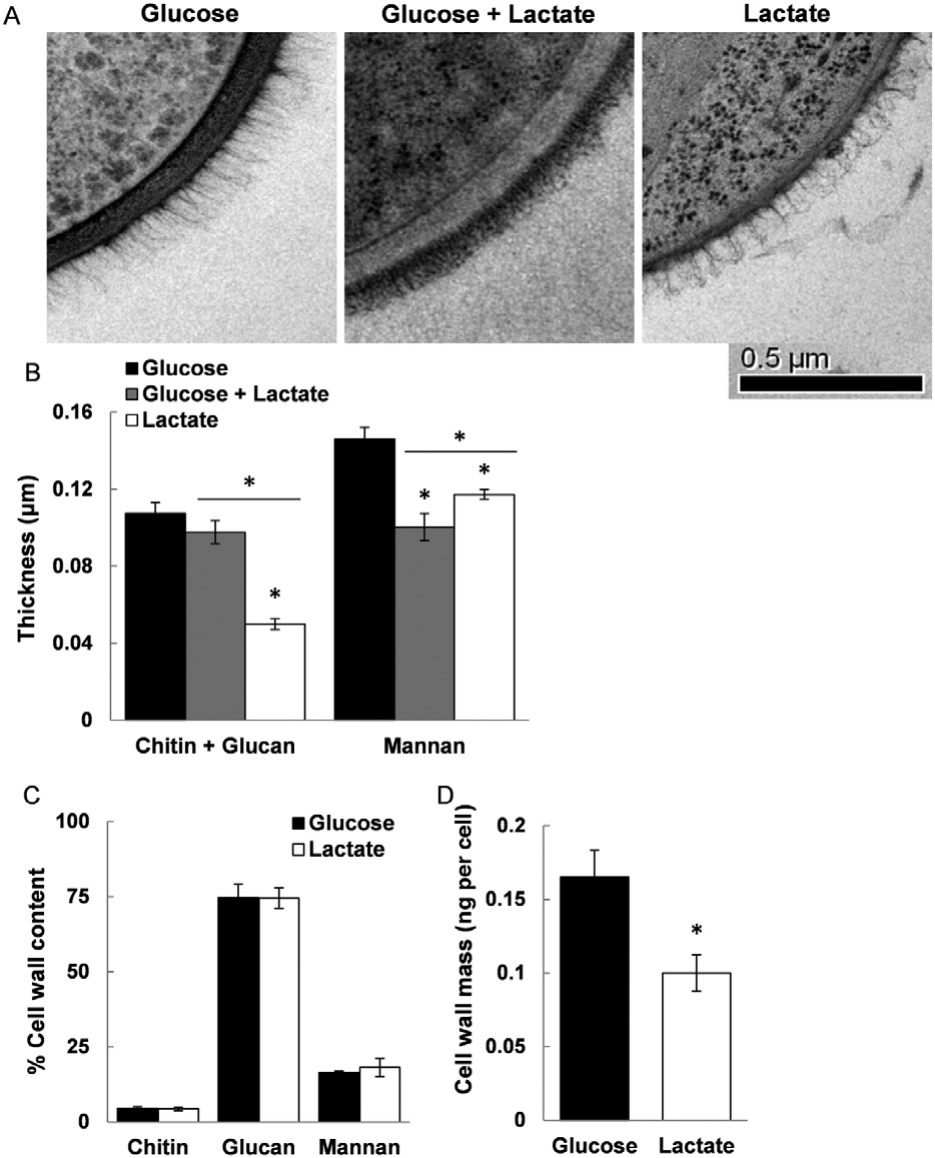
Carbon source influences cell wall architecture. A. TEM images of the *C. albicans* RM1000 cell walls grown on glucose, lactate or a mixture of both (scale bar = 0.5 μm). B. The thicknesses of the chitin plus β-glucan and mannan layers were quantified from TEM images of individual cells. Means ± SEM for *n* > 20 cells for each growth condition are shown. C and D. Biochemical content (C) and cell wall biomass (D) of exponential *C. albicans* cells grown on glucose or lactate. Means ± SEM for three independent experiments are shown. Relative to glucose-grown cells: *P < 0.05.

The cell wall architecture of yeast cells grown on glucose, lactate or a combination of these two carbon sources was examined by high-resolution freeze substitution transmission electron microscopy (FS-TEM). This revealed major differences in cell wall structure, thickness and density (Fig. 1A), which were confirmed by measuring the thickness of β-glucan and mannan layers (Fig. 1B). The cell walls of lactate-grown cells were thinner, with the β-glucan and chitin layer dramatically reduced. Furthermore, the outer mannan fibrils displayed less ordered structures compared with those of glucose-grown cells.

We also examined the effects of carbon source upon *C. albicans* cell wall composition. Lactate- and glucose-grown cells were similar with regard to the overall proportions of chitin, β-glucan and mannan in their cell walls (Fig. 1C). However, there were dramatic differences in cell wall biomass. The cell wall dry mass of lactate-grown cells was 50% less than that of glucose-grown cells (Fig. 1D). Clearly, carbon source strongly influences the cell wall architecture of *C. albicans*.

### Carbon source affects biophysical properties of the cell wall

**Fig. 2:**
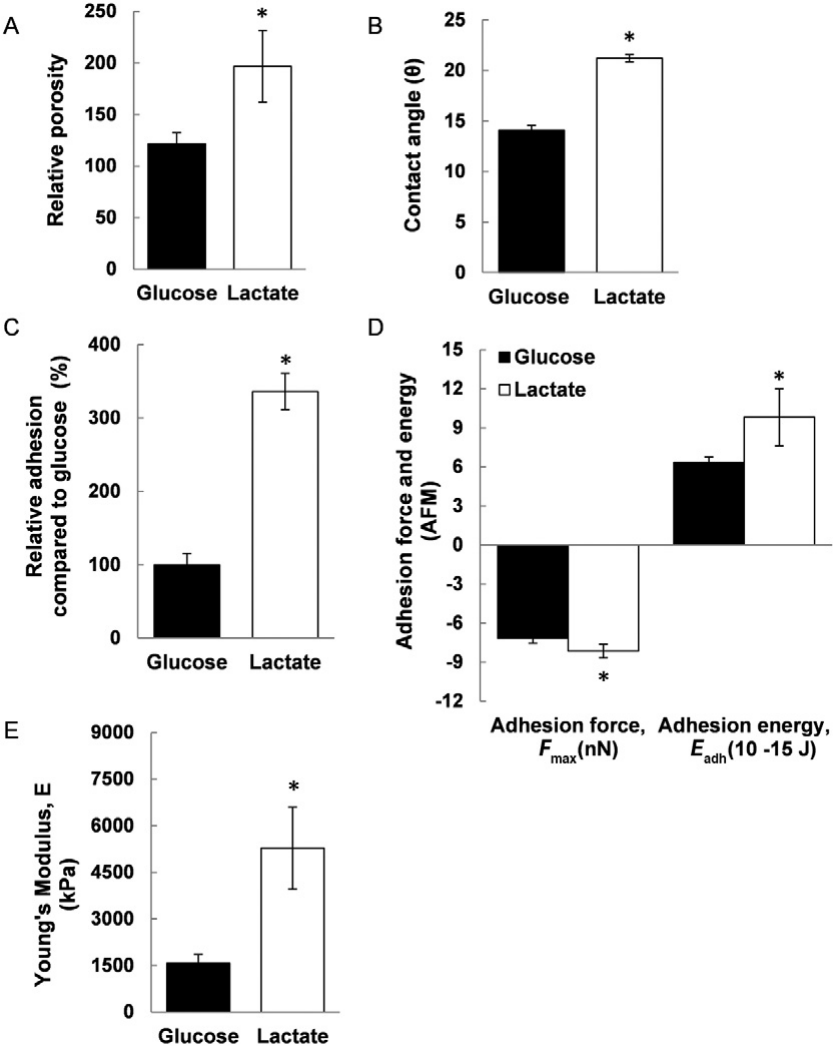
Carbon source affects biophysical properties of the cell wall. *C. albicans* RM1000 cells were grown to mid-exponential phase (OD_600_ 0.4–0.6) prior to the analyses. A. The relative cell wall porosity of glucose or lactate-grown cells assayed via polycation-induced leakage of UV-absorbing compounds (De Nobel *et al*., 1990). B. Cell surface hydrophobicity of glucose- and lactate-grown *C. albicans* RM1000 cells assayed via the water contact angles between a liquid surface and these cells (*n* > 20 drops). C. Adhesion of *C. albicans* cells to a non-treated polystyrene plastic surface. Adhered cells were scraped from the surface, serially diluted, plated on agar and their numbers determined as CFUs (shown as percentage CFUs compared to glucose-grown controls). D. Adhesion force and adhesion energy of glucose- and lactate-grown cells examined by AFM (*n* = 6). E. Cell surface flexibility (indicated by Young's modulus) measured by AFM (*n* = 6). Results are presented as means ± SEM for at least three independent experiments. Relative to glucose-grown cells: *P < 0.05.

Given the major impact on cell wall architecture, we reasoned that changes in carbon source might also affect *C. albicans* cell wall properties. Cell wall porosity was investigated using an assay based on the polycation-induced leakage of UV-absorbing compounds from cells (De Nobel *et al*., 1990). This assay compares leakage induced by small polycations (e.g. poly-l-lysine, which induces cell leakage independent of cell wall porosity) with the release caused by large polycations (such as DEAE-dextrans, which cause limited leakage depending upon the degree of porosity of the cell wall). The cell wall porosity of lactate-grown cells was twofold higher than of glucose-grown cells (Fig. 2A), providing the evidence that carbon source has a major impact upon biophysical properties of the *C. albicans* cell wall. The increased porosity of lactate-grown cells was consistent with their reduced mannan fibrils (Fig. 1B), as increased porosity has been previously associated with shorter mannan side-chains (De Nobel *et al*., 1990).

Cell surface hydrophobicity was examined using a biophysical assay that defines contact angles between cell surfaces and aqueous solutions (Amaral *et al*., 2006; Henriques *et al*., 2007). Significant differences were observed in these contact angles (Fig. 2B) suggesting that the surface of lactate-grown cells is more hydrophobic than that of glucose-grown cells.

Hydrophobicity has been recently linked with adhesion to abiotic surfaces in *C. albicans* clinical isolates (Yoshijima *et al*., 2010; Sardi *et al*., 2011). Adhesion is a key virulence trait, playing major roles in tissue colonization and biofilm formation on indwelling devices in hospital patients (Calderone, 2002). Adhesion of *C. albicans* cells is influenced by growth on different sugars (Willis *et al*., 2000; Bain *et al*., 2001) and therefore we predicted that growth on non-fermentable carbon sources might also influence *C. albicans* adherence. As expected, lactate-grown *C. albicans* cells adhered more strongly to plastic surfaces than cells grown on glucose (Fig. 2C). Furthermore, we confirmed that the adhesive force of lactate-grown cells was significantly greater than for glucose-grown cells using atomic force microscopy in force-mapping mode (AFM-FM) (Müller and Dufrêne, 2007) (Figs 2D and S1). Moreover, the adhesion energy displayed by lactate-grown cells was also significantly elevated (> 50%) (Fig. 2D).

The elasticity of the *C. albicans* cell wall was also examined by AFM. This nanomechanical analysis revealed a threefold increase in Young's modulus (Dague *et al*., 2010) for lactate-grown cells (Fig. 2E), indicating that their cell walls were less elastic than those of glucose-grown cells. Therefore, increased mechanical pressure exerted by lactate-grown cells might promote epithelial penetration during host invasion (Dalle *et al*., 2009).

Taken together, these data indicate that changes in carbon source have a major impact upon the biophysical properties of the cell wall and upon the adherence of *C. albicans* cells, a key virulence trait of this pathogen.

### Osmotic stress resistance and adaptation are affected by carbon source

**Fig. 3:**
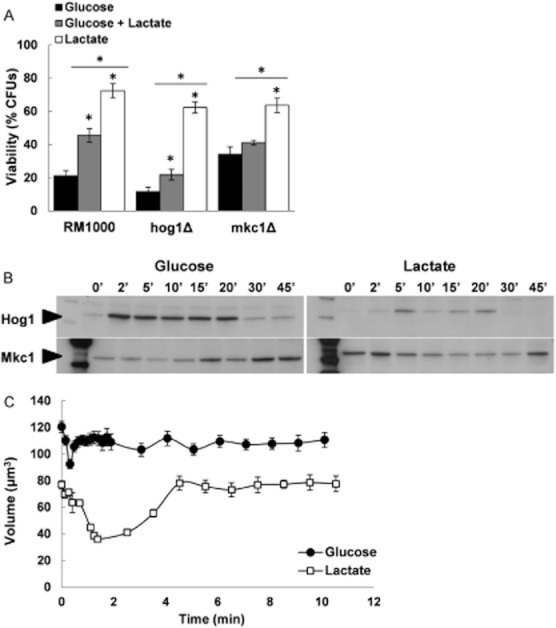
Osmotic stress resistance and adaptation are affected by carbon source. A. Resistance of wild-type (RM1000), *hog1Δ* and *mkc1Δ C. albicans* cells (Table S1) to osmotic stress (2 M NaCl) during exponential growth on glucose, lactate or glucose plus lactate. Means ± SEM for four independent experiments are presented. Relative to glucose-grown cells: *P < 0.05. B. Phosphorylation and activation of Hog1 and Mkc1 in glucose- and lactate-grown cells following exposure to 1 M NaCl as revealed by Western blotting with phospho-specific antibodies. Similar results were obtained in four independent experiments. C. Dynamic changes in cell volume following hyperosmotic shock (1 M NaCl; *n* > 25 cells).

As the cell wall physically protects fungal cells from environmental insults, we reasoned that carbon source might also exert effects upon stress resistance in *C. albicans*. First we examined the impact on osmotic stress resistance, showing that lactate-grown cells were much more resistant to high concentrations of salt (2 M NaCl) than glucose-grown cells (Fig. 3A). Blood glucose and lactate levels are maintained within homeostatic limits. Glucose levels are generally about 3–5 mM (∼ 0.1%) and lactate concentrations are about 1–20 mM (up to 0.2%). Lactate levels vary in mucosal niches with concentrations in the vaginal fluid reaching 110 mM (∼ 1.2%) (O'Hanlon *et al*., 2011). Therefore, we tested a wide range of lactate and glucose concentrations (0.1% to 4%). This revealed that differential osmotic stress sensitivities are observed for cells grown at more physiological levels of these carbon sources (Fig. S2).

This increased resistance might reflect increased signalling via the Hog1 stress-activated protein kinase, because this MAP kinase pathway is critical for osmoadaptation in *C. albicans* (San José *et al*., 1996; Smith *et al*., 2004). This was tested by examining the dynamics of Hog1 phosphorylation during hyperosmotic stress (1 M NaCl; Fig. 3B). Hog1 was activated to a lesser extent in lactate-grown cells, although they were more resistant to 2 M NaCl. Furthermore, this increased resistance was retained in a *hog1Δ* mutant (Fig. 3A). These observations indicate that these effects of carbon source upon osmotic stress resistance were not dependent upon Hog1 signalling.

The cell integrity (Mkc1) signalling pathway also plays a role in cell wall biogenesis and osmoadaptation in *C. albicans* (Navarro-Garcia *et al*., 1998; 2005). Therefore, we examined the dynamics of Mkc1 phosphorylation (Fig. 3C). Higher basal levels of Mkc1 phosphorylation were observed in lactate-grown cells, suggesting active cell wall remodelling even in the absence of stress. While Mkc1 phosphorylation was delayed in glucose-grown cells, Mkc1 activation was observed immediately after NaCl addition to lactate-grown cells (Fig. 3C). However, *MKC1* deletion did not block the effects of lactate on osmotic stress resistance (Fig. 3A). We conclude that the effects of lactate are not dependent on signalling via the cell integrity pathway.

As signalling through these key pathways was not essential for the elevated osmotic stress resistance of lactate-grown cells (Fig. 3A) and given the dramatic changes observed in cell wall architecture (Fig. 1), we reasoned that the increased osmotic stress resistance of lactate-grown cells might be mediated through alterations in the biophysical properties of the cell wall. To test this we examined the dynamic changes in cell volume propagated by hyperosmotic stress (Fig. 3C). Relative to glucose-grown cells, lactate-grown cells displayed dramatic reductions in cell volume (> 50% versus 20%). Furthermore, we observed significant decreases in the rates of volume reduction and recovery in lactate-grown cells (−30.65 μm^3^ min^−1^ and 13.75 μm^3^ min^−1^ respectively) compared to glucose-grown cells (−87.21 μm^3^ min^−1^ and 38.56 μm^3^ min^−1^ respectively). Thus, although the decrease in cell volume was more dramatic for lactate-grown cells, their volume changes occurred at a slower rate, consistent with the AFM observation that the walls of lactate-grown cells are less elastic (Fig. 2E). Taken together, these observations suggest that changes in carbon source affect the architecture and flexibility of the cell wall, and that this in turn affects the dynamics of cellular adaptation and resistance to hyperosmotic stress.

### Carbon source affects resistance to other stresses and antifungal drugs

**Fig. 4:**
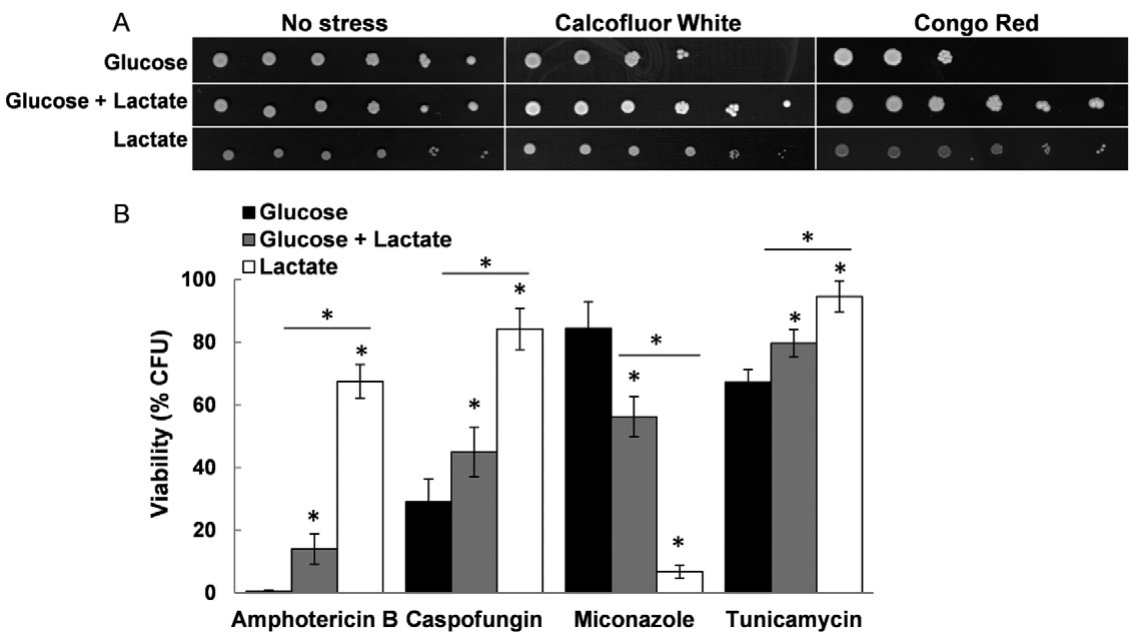
Carbon source impacts upon resistance to other stresses and antifungal drugs. A. Sensitivity of *C. albicans* RM1000 to Calcofluor White (200 μg ml^−1^) and Congo Red (300 μg ml^−1^) when grown on glucose, lactate or a mixture of both. B. Sensitivity of *C. albicans* RM1000 to the antifungal drugs amphotericin B (Ambisome; 10 μg ml^−1^), caspofungin (6.4 ng ml^−1^), miconazole (25 μg ml^−1^) and tunicamycin (4 μg ml^−1^). Relative to glucose-grown cells: *P < 0.05.

Clearly, the influence of cell wall remodelling might extend to other types of stress, and cell wall stresses in particular. Calcofluor White and Congo Red interfere with the synthesis and cross-linking of chitin and glucan respectively. Lactate-grown cells displayed increased resistance to both cell wall agents (Fig. 4A). This increased resistance was also observed for *hog1Δ* and *mkc1Δ* cells grown on lactate (Fig. S3A–C).

Next, we compared the sensitivity of glucose- and lactate-grown cells to antifungal drugs. We tested caspofungin (an echinocandin that inhibits 1,3-β-glucan synthase), tunicamycin (a nucleoside antibiotic that blocks *N*-linked mannosylation), amphotericin B (a polyene that interferes with plasma membrane function by binding to ergosterol) and miconazole (an azole that inhibits ergosterol synthesis). Compared with glucose-grown cells, lactate-grown cells were resistant to caspofungin, tunicamycin and amphotericin B, but were more sensitive to miconazole (Fig. 4B). To address the contrasting effects of carbon source on resistance to membrane-targeting antifungals, we tested the sensitivity of an ergosterol biosynthetic mutant (*erg11Δ*) to amphotericin B and miconazole. Ergosterol levels in *erg11Δ* cells are below detection and consequently this strain displays increased resistance to amphotericin B and azoles (Sanglard *et al*., 2003). Although glucose-grown *erg11Δ* cells displayed increased resistance to miconazole compared to the control strain (CAF4-2) (Fig. S3E), the differential miconazole resistance of glucose- and lactate-grown cells was maintained in both mutant and control strains (Fig. S3D and E). Although sterol levels may differ between glucose- and lactate-grown cells, this does not account for their differential sensitivities to ergosterol-targeting antifungals. This suggests that differential carbon source availability *in vivo*, and the resultant cell wall remodelling, is likely to alter the susceptibility of *C. albicans* cells to therapeutic intervention.

### The observed phenotypes extend to other pathogenic *Candida* species

**Fig. 5:**
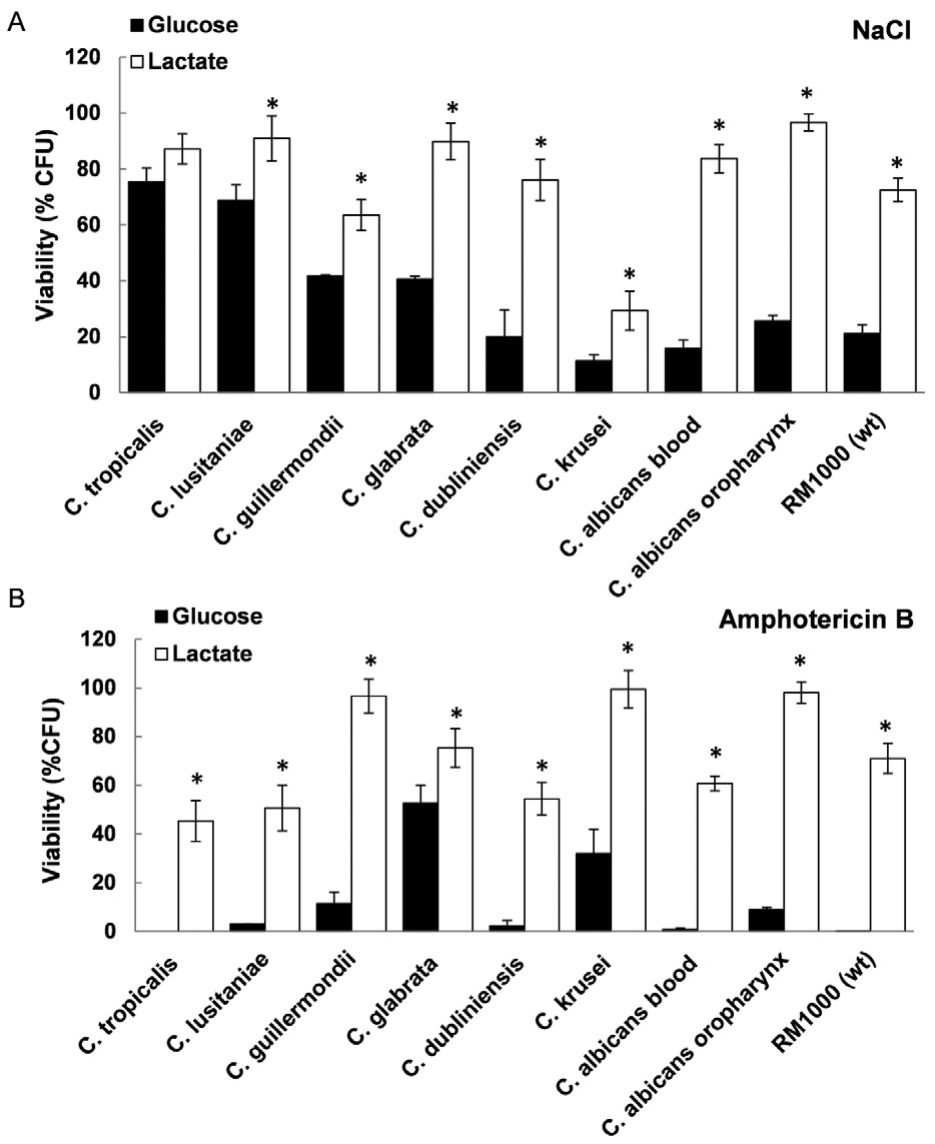
Carbon source impacts upon resistance of other pathogenic *Candida* species. *Candida* cells (Table S1) were grown to exponential phase on glucose or lactate, and their resistance to (A) hyperosmotic stress (2 M NaCl) and (B) amphotericin B (Ambisome; 10 μg ml^−1^) was determined. Means ± SEM for at least three independent experiments are presented. Relative to glucose-grown cells: *P < 0.05.

We tested whether the phenotypic impact of carbon source is observed in other pathogenic *Candida* species. We compared the resistance of lactate- and glucose-grown cells of *C. glabrata*, *Candida dubliniensis*, *Candida tropicalis*, *Candida krusei*, *Candida lusitaniae* and *Candida guillermondii* clinical isolates (Table S1) to hyperosmotic stress and amphotericin B. In addition, we included two *C. albicans* clinical isolates (ATCC90028, a blood isolate, and ATCC10231, an oropharynx isolate) for comparison with the laboratory strains examined in the above experiments (MacCallum *et al*., 2009). In all cases, when these *Candida* species were grown on lactate they displayed significantly increased resistance to hyperosmotic stress and amphotericin B compared with cells grown on glucose (Fig. 5). This confirmed that carbon source has a pronounced effect upon stress and drug resistance for all of the pathogenic *Candida* species tested.

### Observed phenotypes extend to other physiologically relevant carbon sources

Host niches contain complex mixtures of fermentable and non-fermentable carbon sources. Therefore, we extended our analyses to test the impact of mixed carbon sources. Growing *C. albicans* cells on a mixed medium containing both glucose and lactate (each at 1%) resulted in intermediate levels of resistance compared to cells grown on glucose or lactate alone (Figs 3A and 4). This correlated with the intermediate architecture of the cell wall in cells grown on lactate plus glucose (Fig. 1A and B).

**Fig. 6:**
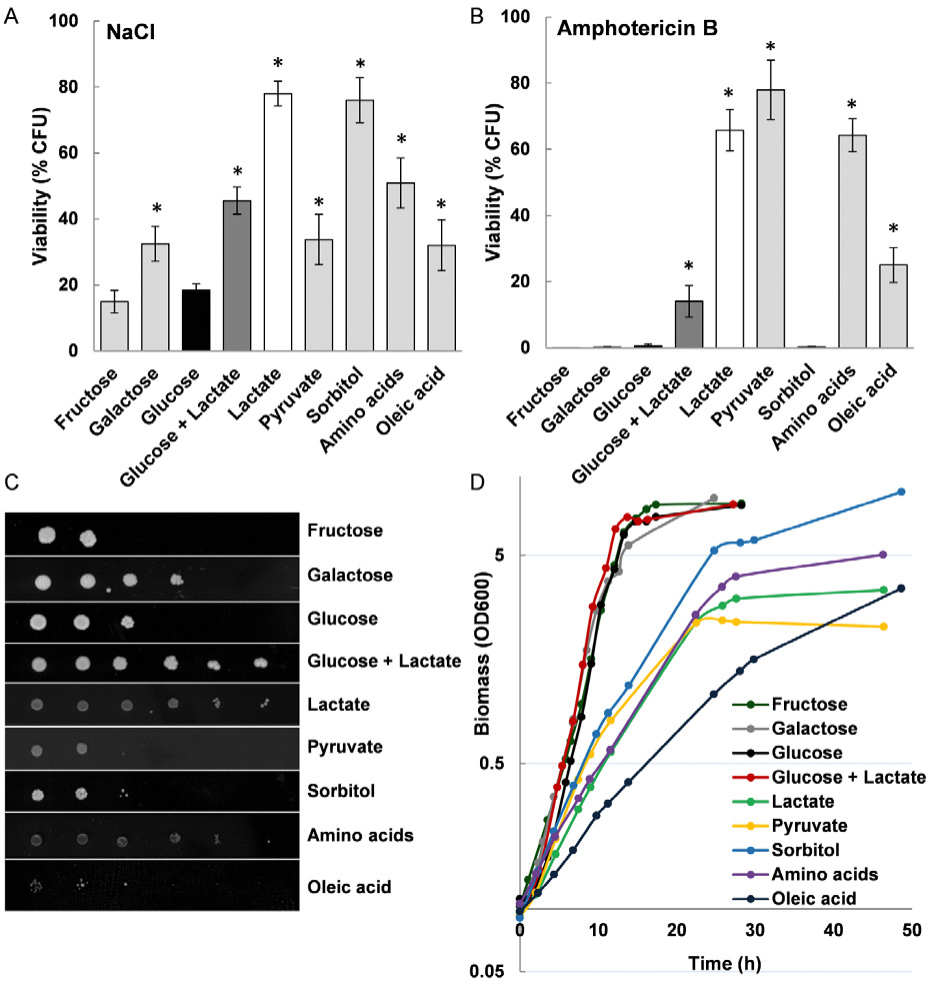
Impact of various carbon sources upon stress and drug resistance. A–C. Sensitivity of *C. albicans* RM1000 to (A) 2 M NaCl, (B) 10 μg ml^−1^ amphotericin B and (C) 300 μg ml^−1^ Congo Red following growth on different carbon sources. Data represent results from at least three independent experiments (means ± SEM). Relative to glucose-grown cells: *P < 0.05. D. Growth curves of *C. albicans* RM1000 on different carbon sources. Cells were grown in either 2% or 0.2% (for oleic acid) carbon source for 4 h at 30°C and the relative levels of growth were determined by monitoring the OD_600_. Each curve represents the average of three biological replicates, with a maximum SEM ± 0.4.

Next, we examined how stress and drug resistance are affected by other carbon sources, including sugars (galactose, fructose and sorbitol), a fatty acid (oleic acid), another short-chain organic acid (pyruvate) and mixed amino acids. Cells grown on these carbon sources displayed significant differences in their sensitivities to hyperosmotic stress, cell wall stress and antifungal drugs when compared to the glucose-grown cells (Fig. 6). In general, cells grown on sugars exhibited lower resistances to osmotic stress (Fig. 6A) and higher sensitivities to amphotericin B (Fig. 6B).

It was conceivable that stress resistance simply correlated with a decreased growth rate. Hence, we monitored the growth of *C. albicans* cells on the different carbon sources during 48 h (Fig. 6D) and calculated the doubling times of these cultures during exponential phase (Fig. S4A). The doubling time was then plotted against the resistance level to hyperosmotic stress (Fig. S4B), amphotericin B (Fig. S4C) and Congo Red (Fig. S4D). This showed that stress resistance did not correlate directly with the relative doubling time on different carbon sources (Spearman correlation coefficients: sensitivities to osmotic stress *P* = 0.46; amphotericin B *P* = 0.062; Congo Red *P* = 0.65; Fig. S3B–D). This was most clearly illustrated by the sensitivities of cells grown in medium containing glucose plus lactate. These cells displayed similar growth rates to those grown on glucose alone, but significant differences were observed in their resistance to osmotic stress, cell wall stresses and antifungal drugs (Figs 3A and 4). Therefore, stress resistance did not correlate inversely with growth rate.

### Growth on serum affects *C. albicans* cell wall architecture

**Fig. 7:**
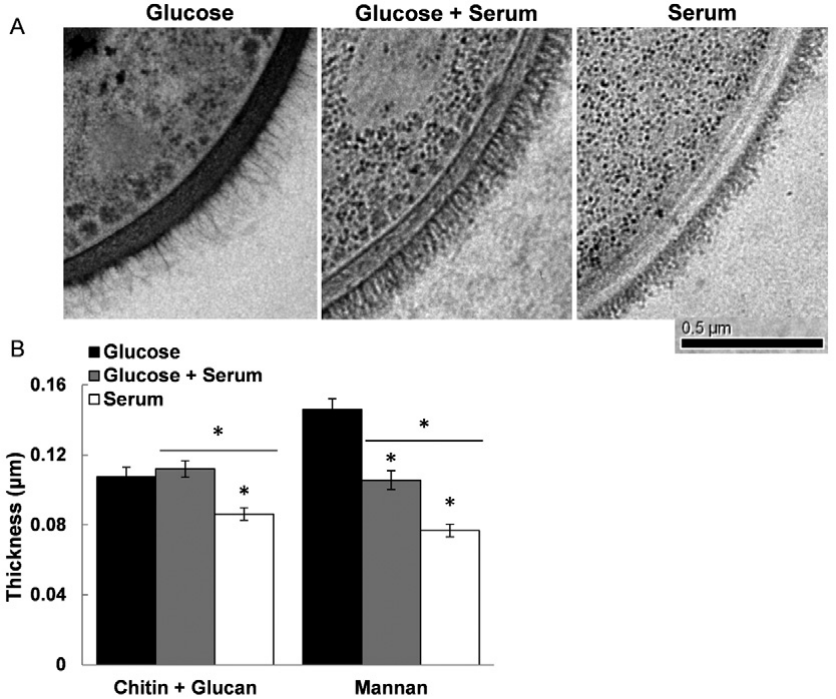
Growth on serum affects cell wall architecture. A. TEM of the *C. albicans* RM1000 yeast cell walls growing on 2% glucose, serum or serum plus 1% glucose (scale bar = 0.5 μm). B. The thicknesses of the chitin plus β-glucan and mannan layers were quantified from TEM images of individual cells. Means ± SEM for *n* > 20 cells for each growth condition are shown. Relative to glucose-grown cells: *P < 0.05.

We analysed mixed carbon sources because host microenvironments contain mixtures of nutrients (above). To reinforce the physiological relevance of our observations, we examined the impact of serum upon cell wall architecture. As expected, the cell walls of *C. albicans* grown in serum (< 0.1% glucose) showed marked differences compared with those of glucose-grown cells (Fig. 7A). In particular, a dramatic decrease in the length of mannan chains and a decrease in the thickness of the chitin and β-glucan layer were observed (Fig. 7B). These differences in the mannan length and structure were consistent with a recent biochemical report describing alterations in mannan complexity in cells grown on blood or serum agar (Kruppa *et al*., 2011). We observed that cells grown on serum supplemented with 1% glucose displayed intermediate phenotypes, marked by a significant lengthening of the outer mannan fibrils (Fig. 7A). *C. albicans* cells grown on serum displayed high levels of filamentation and self-adhesion, thereby precluding colony-forming units (CFUs) measurements. Therefore, we were unable to compare the stress or drug resistance of these cells with lactate- and glucose-grown cells (Figs 3 and 4). Nevertheless, it was clear that serum-grown cells displayed analogous changes in cell wall architecture to those grown on a mixed carbon source (Figs 1A and 7A).

### Carbon source affects the virulence of *C. albicans*

The above observations strongly suggested that changes in carbon source might affect the physiological fitness of *C. albicans* cells *in vivo*. To test this, we assayed the virulence of cells grown on galactose, glucose, oleic acid, lactate, glucose plus lactate or a mix of amino acids in mouse models of systemic and vaginal infection.

**Fig. 8:**
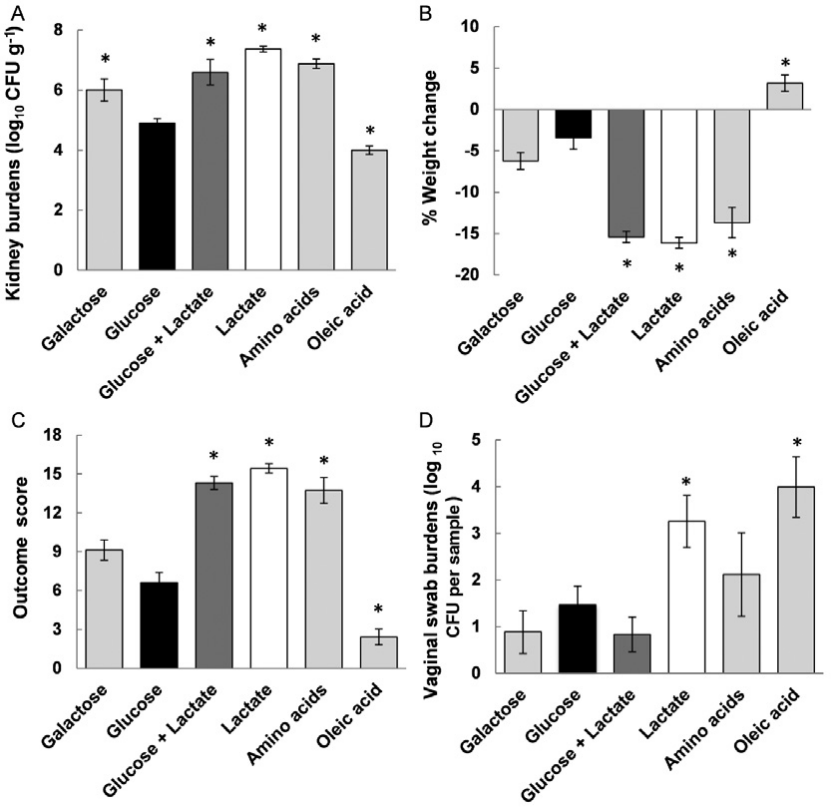
Growth on alternative carbon sources influences *C. albicans* virulence. *C. albicans* (RM1000 containing Clp20, Table S1) cells grown on different carbon sources were used to infect mice intravenously. A and B. Kidney burdens (A) and weight change (B) were measured 72 h post infection (means ± SEM; *n* = 6). C. Infection outcome scores were calculated after 72 h (means ± SEM; *n* = 6), higher outcome scores reflecting more severe infection (MacCallum *et al*., 2010). D. Mice were infected intravaginally with *C. albicans* (RM1000 containing Clp20) cells grown on different carbon sources. Fungal burdens in vaginal swabs were measured 72 h post infection (means ± SEM; *n* = 6). In all panels, the asterisk (*) indicates *P* < 0.05 relative to glucose-grown *C. albicans* infection.

For systemic infection, virulence was assayed by determining infection outcome scores, which are based on measurements of weight changes and kidney burdens 3 days post challenge (MacCallum *et al*., 2010). In this assay a higher ‘outcome score’ reflects greater weight loss and higher fungal burdens and thus increased virulence (MacCallum *et al*., 2010). Carbon source was found to have a significant impact upon the virulence of *C. albicans* during systemic infection (Fig. 8). Indeed, significant differences were found for all parameters (kidney burden, weight change and outcome score). Mice infected with *C. albicans* cells grown on lactate, glucose plus lactate, amino acids all displayed increased fungal burdens and weight loss compared with glucose-grown cells (Fig. 8A and B). In contrast, mice infected with *C. albicans* cells grown on oleic acid displayed reduced fungal burdens in the kidney and these mice actually gained weight (Fig. 8A and B). Therefore, growth on oleic acid reduced the virulence of *C. albicans* in systemic infections (Fig. 8C). These observations closely paralleled the stress phenotypes induced by growth on alternative carbon sources (Fig. 6).

For vaginal infection, virulence was assayed by measuring fungal burdens in mice 3 days post infection. Significantly increased virulence was observed for *C. albicans* cells grown on lactate or oleic acid compared to the control glucose-grown cells (Fig. 8D). Differences for the other carbon sources were not statistically significant. Interestingly, growth on oleic acid led to reduced virulence in the intravenous model but increased virulence in the vaginal model (Fig. 8C and D).

## Discussion

*Candida albicans* occupies diverse niches in its human host and many of these microenvironments offer complex mixtures of nutrients. Some niches continuously supply low concentrations of glucose (e.g. ∼ 0.1% in the blood, during haematogenously disseminated candidiasis), while others provide transient exposure to fermentable sugars (e.g. the oral cavity and gastrointestinal tract) or probably lack glucose (e.g. on skin or nails). Nevertheless, it is often assumed that *in vitro* observations made with cells grown on relatively high concentrations of glucose are relevant to growth *in vivo*. Almost without exception, the experimental dissection of fitness attributes, such as stress adaptation, in *C. albicans* has been performed on glucose-grown cells (Alonso-Monge *et al*., 2003; Enjalbert *et al*., 2003; Smith *et al*., 2004; Navarro-Garcia *et al*., 2005; Eisman *et al*., 2006; Cheetham *et al*., 2007). Furthermore, antifungal drug susceptibilities are generally assayed on glucose-grown cells, the glucose concentration and the medium composition significantly influencing the minimum inhibitory concentration of drug (Bartizal and Odds, 2003).

We reveal that growth on alternative, physiologically relevant carbon sources has a major impact upon the stress, drug resistance and virulence of *C. albicans*. Cells grown on some non-fermentable carbon sources were more resistant to amphotericin B and caspofungin as well as to osmotic and cell wall stresses (Figs 3, 4 and 6). These observations were replicated in other pathogenic *Candida* species (Fig. 5). Consistent with these *in vitro* observations, growth of *C. albicans* cells on alternative carbon sources significantly altered their virulence (Fig. 8). Moreover, the impact on virulence was highly dependent on the site of infection. While growth on some carbon sources (e.g. oleic acid) promoted virulence during mucosal infection, presumably conferring a fitness advantage during disease onset in this environment, the same carbon source reduced virulence in a systemic infection (Fig. 8). Interestingly, growth on sugars such as glucose or galactose resulted in low fungal burdens in both infection models, whereas growth on amino acids or lactate promoted both systemic and vaginal infection. A recent study has also suggested that lactate utilization is required for *Candida* proliferation in the gastrointestinal tract (Ueno *et al*., 2011).

It has been reported previously that the growth medium used to prepare *C. albicans* inoculums affects the ability of strains to gain an initial invasive advantage immediately after injection into host, therefore influencing virulence (Odds *et al*., 2000). Our data now indicate that the carbon source strongly influences cell wall architecture and environmental adaptation during infection. Although *C. albicans* cells will adapt to the available nutrients in their local niche (Lorenz *et al*., 2004; Rodaki *et al*., 2009), our data show clearly that the initial fitness advantage conferred by growth on different carbon sources is ultimately reflected in the infection outcome in both systemic and vaginal infections (Fig. 8). Obviously differential carbon source availability within diverse host niches will also influence the virulence of *C. albicans* during disease progression.

It was conceivable that carbon source might influence stress sensitivity in *C. albicans* via key stress signalling mechanisms such as the Hog1 and the Mkc1 pathways. However, elevated stress resistance did not correlate with increased activation of the Hog1 and Mkc1 MAP kinases (Fig. 3B). Moreover, the increased resistance of lactate-grown cells was not blocked by inactivation of either kinase (Fig. 3A). Therefore, while the impact of carbon upon signalling almost certainly contributes to stress adaptation, this does not account for the major effects of carbon source upon stress resistance.

Instead, carbon source exerts its effects on stress resistance largely through alterations in the *C. albicans* cell wall. Growth on a non-fermentable carbon source, or mixtures of fermentable plus non-fermentable carbon sources, led to major changes in the *C. albicans* cell wall architecture (Fig. 1). These changes were manifested in significant alterations in the biophysical and mechanical properties of the cell wall, some of which affect stress adaptation (Fig. 2). Such properties have been predicted by mathematical modelling to play an important role in the adaptive processes of yeast cells (Schaber *et al*., 2010). Therefore, we suggest that the observed changes in cell wall elasticity led to decreased rates of cell volume change in *C. albicans* cells (Fig. 3C) thereby promoting their increased osmotic stress resistance. Presumably, the major remodelling of the cell wall in response to carbon source also underlies the altered cellular resistance to cell wall stresses and antifungal drugs.

How does the carbon source influence the cell wall? Given that remarkably little is known about how the cell wall polysaccharides are interlinked, the detailed mechanisms by which carbon source affects these linkages may take some time to define. However, the carbon source is likely to affect cell wall biogenesis by impacting directly upon metabolic fluxes as well as indirectly through regulatory networks (Ihmels *et al*., 2005; Martchenko *et al*., [Bibr b42]; Askew *et al*., 2009). We reason that when glucose is present at high concentrations, excess carbon might flow via hexose phosphates into the biosynthesis of β-glucan and mannan, generating an elaborate cell wall that is relatively thick. In contrast, during growth on non-fermentable carbon sources the hexose phosphates required for cell wall biosynthesis must be generated by gluconeogenesis, an energetically demanding process. Less carbon is probably committed to cell wall biosynthesis under these more challenging conditions, leading to the construction of a leaner but stiffer cell wall.

In conclusion, our findings show clearly that carbon source significantly influences the resistance of *C. albicans* and other pathogenic *Candida* species to environmental stresses and antifungal drugs. This infers that carbon sources such as lactate and amino acids, which are available in many human niches, modulate the fitness of *C. albicans* cells *in vivo*, and furthermore that differential nutrient availability within diverse host niches has a strong influence upon the ability of *Candida* cells to counteract local stresses and resist pharmacological intervention. Indeed, growth on different carbon sources strongly influenced the virulence of *C. albicans* in murine models of systemic and vaginal candidiasis. The next challenge is to define which local nutrients are assimilated by *C. albicans* cells during disease establishment and progression, and how this influences the behaviour of this pathogen in the host.

## Experimental procedures

### Strains and growth conditions

The *Candida* strains and clinical isolates used in this study are described in Table S1. Strains were grown at 30°C in minimal medium containing 2% carbon source (glucose, fructose, galactose, sodium lactate, sodium pyruvate, sorbitol or a mix of amino acids), 0.67% yeast nitrogen base without amino acids (YNB) and supplemented with 10 μg ml^−1^ of the appropriate auxotrophic requirements. Similar to previous analyses (Piekarska *et al*., 2006), a lower concentration of carbon source was used for cells grown on oleic acid (0.2% instead of 2%). For serum experiments, cells were grown in 20% fetal bovine serum in PBS (Invitrogen, Paisley, UK) with or without 1% glucose. Unless otherwise stated, RM1000 (Negredo *et al*., 1997) cells were grown overnight at 30°C, diluted to an OD_600_ of 0.1 in fresh medium, and harvested in mid-exponential phase (OD_600_ = 0.5) for analyses and sensitivity assays. With the exception of serum cultures (pH 6.5–7.0), all experiments were performed with yeast cells grown at a pH 5.2–5.6.

Growth was monitored on different carbon sources by measuring the biomass of cultures (OD_600_) during 48 h of growth. Each curve represents the average of three biological replicates, with a maximum SEM ± 0.4. The linear equation during exponential phase was calculated for each curve, and the doubling time was calculated using this equation.

### Freeze substitution transmission electron microscopy

Freeze substitution transmission electron microscopy was performed as described previously (Netea *et al*., 2006), except that *C. albicans* cells were harvested by filtration rather than by centrifugation to maximize cell wall integrity and ultrathin sections were cut at a thickness of 100 nm. Samples were imaged with a Philips CM10 transmission microscope (FEI UK) equipped with a Gatan 600 W camera and images were recorded using Digital Micrograph (Gatan, Abingdon Oxon, UK). The thicknesses of the chitin plus β-glucan and mannan cell wall layers were measured using Image J and by averaging > 20 measurements for each cell (*n* > 20 cells).

### Cell wall biochemistry

The chitin, β-glucan and mannan content of cell walls were measured using previously described procedures (Plaine *et al*., 2008; Lee *et al*., 2011). Briefly, cell walls were prepared from mid-exponential *C. albicans* grown on glucose or lactate. Cells were washed five times with dH_2_O, disrupted with glass beads (Sigma, G9268) using a Fastprep cell breakage machine (Thermo Savant, Middlesex, UK). Extracts were centrifuged at 13 000 r.p.m. for 3 min and washed five times in 1 M NaCl. Cell walls were washed by heating at 100°C for 10 min in 2% SDS, 0.3 M β-mercaptoethanol, 1 mM EDTA, 50 mM Tris-HCl, pH 6.8. Cell wall pellets were then washed and resuspended in dH_2_O, freeze-dried and the dry weight recorded to calculate the cell wall biomass per cell. The cell wall pellets were hydrolysed in 100% trifluoroacetic acid at 100°C for 3 h, the acid evaporated at 90°C for 1 h and samples resuspended in sterile dH_2_O at 10 mg ml^−1^. Glucosamine, glucose and mannose contents were then determined by high-performance anion-exchange chromatography with pulsed amperometric detection (HPAEC-PAD) in a carbohydrate analyser system from Dionex (Surrey, UK) (Lee *et al*., 2011), using hydrolysed samples from three independent biological replicates, each with technical duplicates.

### Cell wall porosity

Cell wall porosity was assayed using methods adapted from an existing protocol (De Nobel *et al*., 1990). Briefly, mid-exponential cells were washed twice with dH_2_O and aliquots of 10^8^ cells were incubated with shaking (200 r.p.m.) for 30 min at 30°C in 1 ml of either 10 mM Tris-HCl pH 7.4 (control), the same buffer containing 5 mg ml^−1^ DEAE-dextran (500 kDa) or buffer containing 15 μg ml^−1^ poly-l-lysine (50 kDa). Cells were then pelleted by centrifugation and the A_260_ of supernatants measured. Relative porosity was calculated using the following formula: relative porosity = 100 × (A_DEAE_ − A_buffer_) / (A_poly-l-lysine_ − A_buffer_). Results are means ± SEM of five independent experiments, each with two technical replicates.

### Surface hydrophobicity

Microscope slides were prepared with a uniform layer of 2% agar in 10% glycerol in water. *C. albicans* cells were harvested in mid-exponential phase, resuspended in sterile H_2_O to an OD_600_ of 2 and applied to the surface of these agar slides. This procedure was repeated 2–3 times and the slides left to dry until a uniform lawn was obtained. Water contact angles were then determined at room temperature using the sessile drop technique (Soumya *et al*., 2011) with a goniometer (FTA 1000 Analyzes System, First Ten Angstroms, Cambridge, UK) according to manufacturer's instructions. At least seven independent measurements were taken for each slide and three independent replicates were analysed for each growth condition.

### Cell adhesion

Mid-exponential *C. albicans* cells were washed twice with dH_2_O and resuspended in PBS. About 10^7^ cells in 2 ml of PBS were added to 12-well plates (non-treated polystyrene; Costar, Corning, Ewloe, UK) and cells allowed to adhere for 1 h at 37°C without shaking. After washing three times with PBS, adhered cells were scraped off the plastic surface into 1 ml of PBS and quantified by OD_600_ and counting CFUs. Results represent the average CFUs ± SEM for five independent replicate experiments for each growth condition, each with technical duplicates.

### Atomic force microscopy measurements

The nanomechanical properties (i.e. adhesive force, adhesive energy and Young's modulus) of mid-exponential *C. albicans* cells grown in glucose or lactate were investigated by AFM force mapping (Adya and Canetta, 2011). These experiments were conducted in contact mode and a Si_3_N_4_ cantilever (Bruker UK) of nominal spring constant 0.01 N m^−1^ and a resonant frequency of ∼ 7 kHz was used. The tip (radius of curvature < 10 nm) microfabricated on the AFM cantilever was brought into contact with the surface of the cell. AFM experiments were performed over a grid of 10 × 10 points on an individual cell and the force spectroscopy curves recorded on each point. Measurements were again repeated on three randomly chosen cells per duplicate slide, thus giving a total of six measurements growth condition. During the force mapping experiments an average normal force of 2 nN was applied by the AFM cantilever onto the cell. The tip was withdrawn vertically away from the cell surface at a speed of 0.5 μm s^−1^. In each experiment, the maximum adhesive force in nanonewtons (nN) of the cell surface to the tip (*F*_max_), the maximum distance in micrometres (μm) of deformation of the cell (*d*_max_) and the adhesion energy in joules (J) (*W*_adh_) were measured. The force spectroscopy curves obtained during an AFM force mapping experiment were force–height curves. To transform these curves into force–distance (*F*–*d*) curves, the real distance, *d*, between the sample and the AFM tip was calculated by subtracting the deflection of the cantilever, *z*, from the height values that corresponded to the measured piezodisplacement, *z*_piezo_: *d* = *z*_piezo_ − *z* (Binning *et al*., 1986). The Young's modulus of elasticity (E) was determined by fitting the slope of the trace curve to the Hertz model (Hertz, 1986).

### Cell volume

Mid-exponential *C. albicans* cells were adhered to a poly-l-lysine-coated surface for 30–60 min and stained with 2 μg ml^−1^ Calcofluor White to visualize cell wall chitin. Dynamic changes in individual cell volumes were monitored microscopically using a Zeiss Observer Z.1 microscope (Zeiss, Welwyn Garden City, UK) and their initial volume was recorded using Volocity software (Cambridge, UK). Cells were exposed to 1 M NaCl and changes in cell volume monitored by taking *Z*-stacked images every 10 s for the first minute and then every minute for the next 30 min (*n* > 25 cells for each condition).

### Western blotting

Protein extracts were prepared from mid-exponential *C. albicans* cells and subjected to Western blotting as described before (Smith *et al*., 2004). Hog1 activation was detected using a phospho-specific phospho-p38 MAPK (Thr180/Tyr182) antibody 9211 (New England Biolabs, Hitchin, UK). Mkc1 activation was detected using a phospho-specific phospho-p44/42 MAPK (Erk1/2; Thr202/Tyr204) antibody 4370 (New England Biolabs). In both cases the secondary antibody was HRP-labelled anti-rabbit IgG 7074 (New England Biolabs) which was detected using Pierce ECL PlusTM Western blotting reagents (Thermo Scientific, Cramlington, UK).

### Stress resistance

To examine hyperosmotic stress, mid-exponential *C. albicans* cells were exposed to 2 M NaCl for 1 h, at 30°C and CFUs measured relative to untreated control cells. Means ± SEM for at least five independent experiments are presented. To examine cell wall stresses, serial dilutions of mid-exponential *C. albicans* cells were plated onto agar starting from a dilution of 5 × 10^7^ cells per spot and diluted 1/10 thereafter. The YNB agar contained the specified carbon source and was supplemented with Congo Red (300 μg ml^−1^) or Calcofluor White (200 μg ml^−1^). Plates were incubated for 2–4 days at 30°C and then photographed. Results were compared against no stress plates. Results shown are representative of data accumulated from at least three independent experiments.

### Antifungal susceptibility

Antifungal drug susceptibility was analysed by treating mid-exponential *C. albicans* cells grown on the specified carbon source with tunicamycin (4 μg ml^−1^), caspofungin (0.08 μg ml^−1^), amphotericin B (Ambisome 10 μg ml^−1^ and 20 μg ml^−1^ for two *Candida* pathogenic isolates that showed decreased sensitivity) or miconazole (25 μg ml^−1^) for 1 h at 30°C. Cells were then serially diluted and plated onto agar. CFUs were quantified and antifungal drug sensitivities calculated relative to those observed for untreated cells. Means ± SEM for at least three independent experiments are presented.

### *Candida albicans* virulence assays

All animal experimentation conformed to the requirements of the UK Home Office legislation and of the Ethical Review Committee of the University of Aberdeen.

The 3 day murine intravenous challenge model of *C*. *albicans* infection (MacCallum *et al*., 2010) was used to determine the impact of carbon source on systemic virulence. Female BALB/c mice [6–8 weeks; 18.9 ± 0.1 g (mean ± SEM); Harlan, UK] were randomly assigned to and housed in groups of six with food and water provided *ad libitum*. RM1000 + Clp20 (JC21) (Smith *et al*., 2004) cells were grown overnight at 30°C in YNB medium with 2% carbon source with constant agitation. The cultures were diluted to an OD_600_ of 0.1 in fresh medium, and harvested at mid-exponential phase (OD_600_ = 0.5). Cells were harvested, washed with sterile saline and the cell counts adjusted by OD_600_ to provide a 100 μl cell suspension estimated to deliver a challenge dose of 5 × 10^4^ CFU g^−1^ mouse body weight. Actual challenge doses were determined from viable counts read after 24 h and were approximately 4.8 × 10^4^ CFU g^−1^ (range: 4.2–6.4 × 10^4^ CFU g^−1^). Each inoculum was used to infect one group of mice, which was randomly allocated. Mice were infected intravenously via a lateral tail vein, and were monitored and weighed daily. At 72 h post challenge, mice were weighed, humanely terminated by cervical dislocation, the kidneys removed aseptically and renal fungal burdens determined. For each animal, fungal burdens were measured for both kidneys by viable count. Virulence was assessed by fungal kidney burdens and by percentage weight change at 72 h and an outcome score calculated (MacCallum *et al*., 2010). The outcome score is devised to allow approximately equal contributions of the two parameters and was calculated using the following formula: outcome score = log CFU g^−1^ kidney − (0.5 × percentage weight change) (MacCallum *et al*., 2010). Statistical differences between body weight changes, kidney burdens and outcome scores were determined by the Mann–Whitney and Kruskall–Wallis tests using IBM spss (version 19).

The murine vaginal infection model (Fidel and Sobel, 1999) was used to determine the effect of carbon source on the ability of *C. albicans* to colonize/infect vaginal tissue. Female BALB/c mice [6–8 weeks; 20.2 ± 0.1 g (mean ± SEM); Harlan, UK] were randomly allocated into groups of six mice. On day 3, relative to infection, mice were treated subcutaneously with 20 μg of oestradiol valerate (Sigma) in sesame oil (Sigma). Oestradiol was administered every 3 days during the infection. *C. albicans* inocula were prepared from the cultures used for the intravenous challenge model, aiming for 1 × 10^6^ CFU per 20 μl. Actual inoculum levels were determined by viable counts and were approximately 1 × 10^6^ CFU per mouse (range: 0.9–1.3 × 10^6^ CFU per mouse). Inocula were administered intravaginally using a pipette. Mice were sampled using swabs on day 3 and day 7 post infection. Swabs were dispersed in 100 μl of 3% Tween80 in saline and dilutions plated out on Sabouraud dextrose plus chloramphenicol and gentamicin agar. On day 7 mice were humanely terminated by cervical dislocation.

### Statistical analyses

Results from independent replicate experiments are expressed as means ± SEM. With the exception of animal experiments, results were compared using two-sample Student's *t*-test, with a significance cut-off of 0.05. A significance cut-off of 0.1 was used for adhesion force and adhesion energy measured by AFM. Correlation was determined using the Spearman correlation coefficient (*ρ*) with *P*-values smaller than 0.05 denoting a significant correlation.

## Supporting information

**Fig. S1.** Illustration of the atomic force microscopy experiment (adapted from Adya and Canetta, 2011).

A. Contact between the AFM probe and the sample surface is followed by cantilever bending with AFM probe indentation during the trace (approach) cycle of the AFM experiment. On the retrace (retract) cycle, the sample surface is then stretched until the point of detachment.

B. An example of a typical experimental *F*–*d* curve for a trace and retrace cycle is presented; the stages are identified by the arrows. The meanings of *F*_max_, *d*_max_ and *W*_adh_ are also illustrated.

**Fig. S2.** The impact of carbon source on stress resistance is not dependent on the concentration of the carbon source. Resistance of wild-type (RM1000) *C. albicans* cells to osmotic stress (2 M NaCl) during exponential growth on different concentrations of glucose or lactate (0.1–4%). Means ± SEM for four independent experiments are presented. Relative to glucose-grown cells: **P* < 0.05. Each concentration was also compared against its corresponding glucose or lactate 2% standard concentration: ^#^*P* < 0.05.

**Fig. S3.** The impact of carbon source upon cell wall stress resistance is not dependent on Hog1, Mkc1 signalling or cellular ergosterol levels. Increased resistance to (B) Calcoflouor White (200 μg ml^−1^) and (C) Congo Red (300 μg ml^−1^) was observed in wild-type (RM1000), *hog1Δ* and *mkc1Δ* cells (Table S1) grown on lactate when compared to glucose-grown cells and relative to no-stress plates (A). Plates were incubated for 2–4 days at 30°C and then photographed. Pictures shown are representative of three independent experiments. (D) Resistance to amphotericin B (10 μg ml^−1^) and (E) resistance to miconazole (25 μg ml^−1^) of ergosterol depleted *erg11* cells and corresponding wild-type strain. Relative to glucose-grown cells: **P* < 0.05. The resistance of the *erg11Δ* strain was compared to the corresponding wild-type control, ^#^*P* < 0.05.

**Fig. S4.** Stress resistance does not correlate inversely with growth rate.

A. Doubling times were calculated as the interval of time required by the cells to double from an OD_600_ = 0.5 to 1.0. The linear equation of the exponential phase slope was used for these calculations.

B–D. (B) Resistance to osmotic stress, (C) resistance to amphotericin B and (D) resistance to Congo Red of cells grown on different carbon sources were plotted against their relative doubling times under each growth condition. Resistance to Congo Red was assessed based on the number of dilutions (out of six plated) growing on Congo Red supplemented plates (Fig. 6C). No correlation was found between the rate of growth and level of resistance against the different stressors – Spearman correlation coefficient for (B) NaCl (2 M; *P* = 0.46), (C) amphotericin B (Ambisome; 10 μg ml^−1^; *P* = 0.062) and (D) Congo Red (300 μg ml^−1^; *P* = 0.65).

**Table S1.** List of fungal strains and clinical isolates used in this study.
