# Supplementary material for: Host carbon sources modulate cell wall architecture, drug resistance and virulence in a fungal pathogen
Source: Cell Microbiol. 2012 Jun 5;14(9):1319–35. doi: 10.1111/j.1462-5822.2012.01813.x (PMC3465787; doi:10.1111/j.1462-5822.2012.01813.x)
Supplement: Supplementary file 1 [file cmi0014-1319-SD1.doc]

**Supporting Information**

**
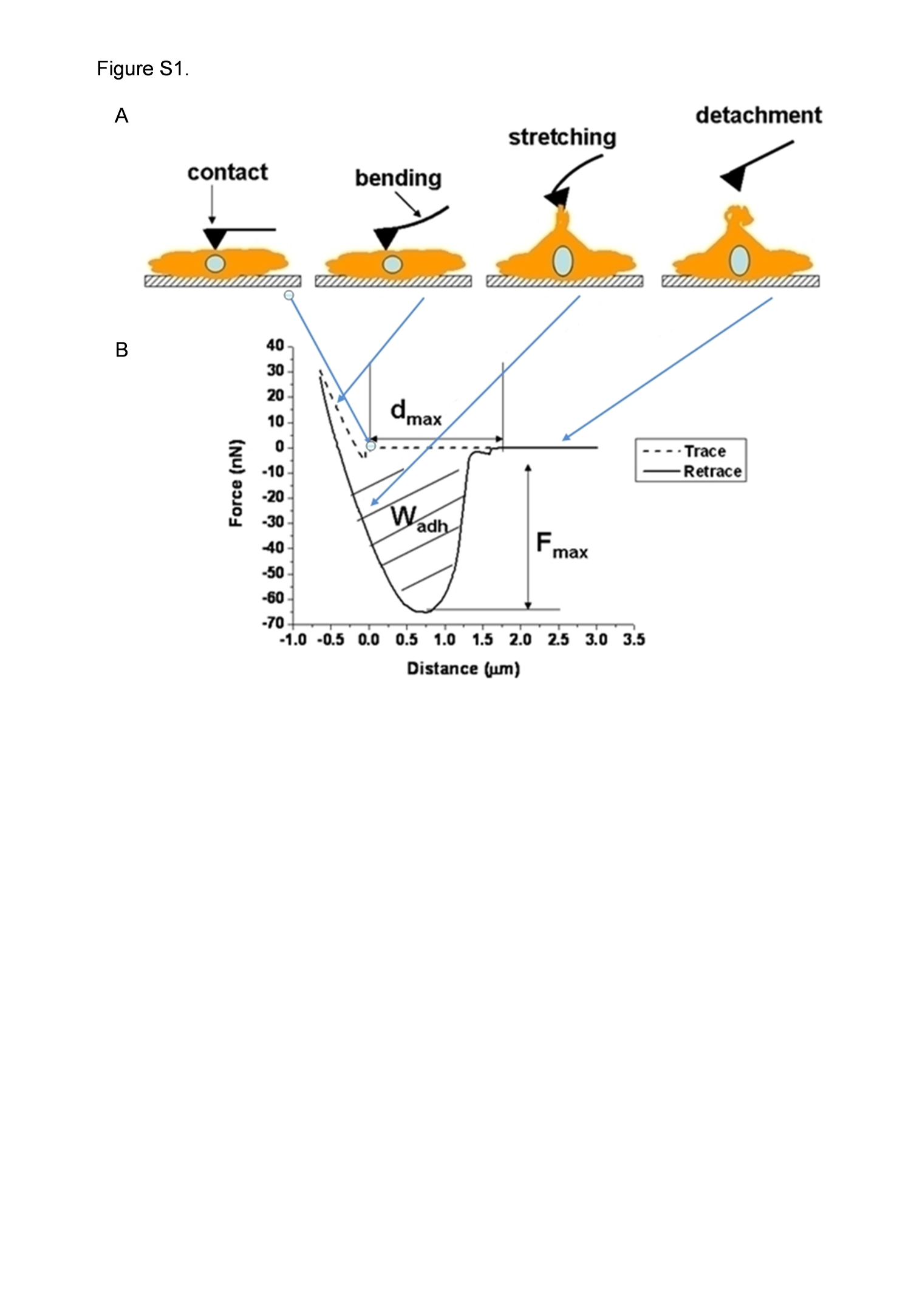
**

**Figure S1**. Illustration of the Atomic Force Microscopy experiment(adapted from AdyaandCanetta, 2011)**.**(A)Contact between the AFM probe and the sample surface is followed by cantilever bending with AFM probe indentation during the trace (approach) cycle of the AFM experiment. On the retrace (retract) cycle, the sample surface is then stretched until the point of detachment. (B) An example of a typical experimental *F-d* curve for a trace and retrace cycle is presented; the stages are identified by the arrows. The meanings of *F_max_*, *d_max_*, and *W_adh_* are also illustrated.


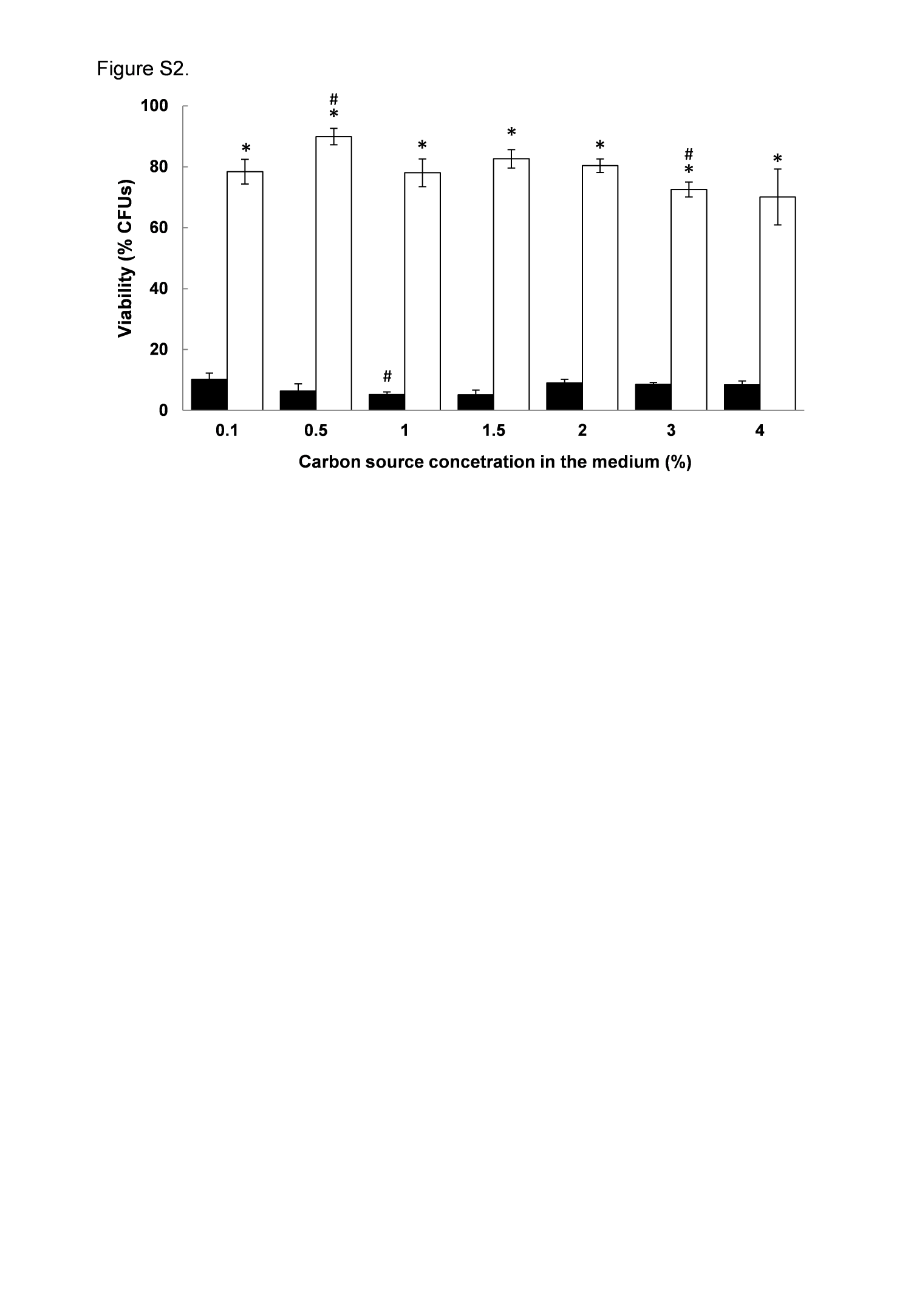


**Figure S2.** The impact of carbon source on stress resistance is not dependent on the concentration of the carbon source. Resistance of wild type (RM1000)*C. albicans*cells to osmotic stress (2 M NaCl) during exponential growth on different concentrations of glucose or lactate (0.1 - 4%). Means ± S.E.M. for 4 independent experiments are presented. Relative to glucose-grown cells: **P*< 0.05. Each concentration was also compared against its corresponding glucose or lactate 2% standard concentration: # *P*< 0.05.


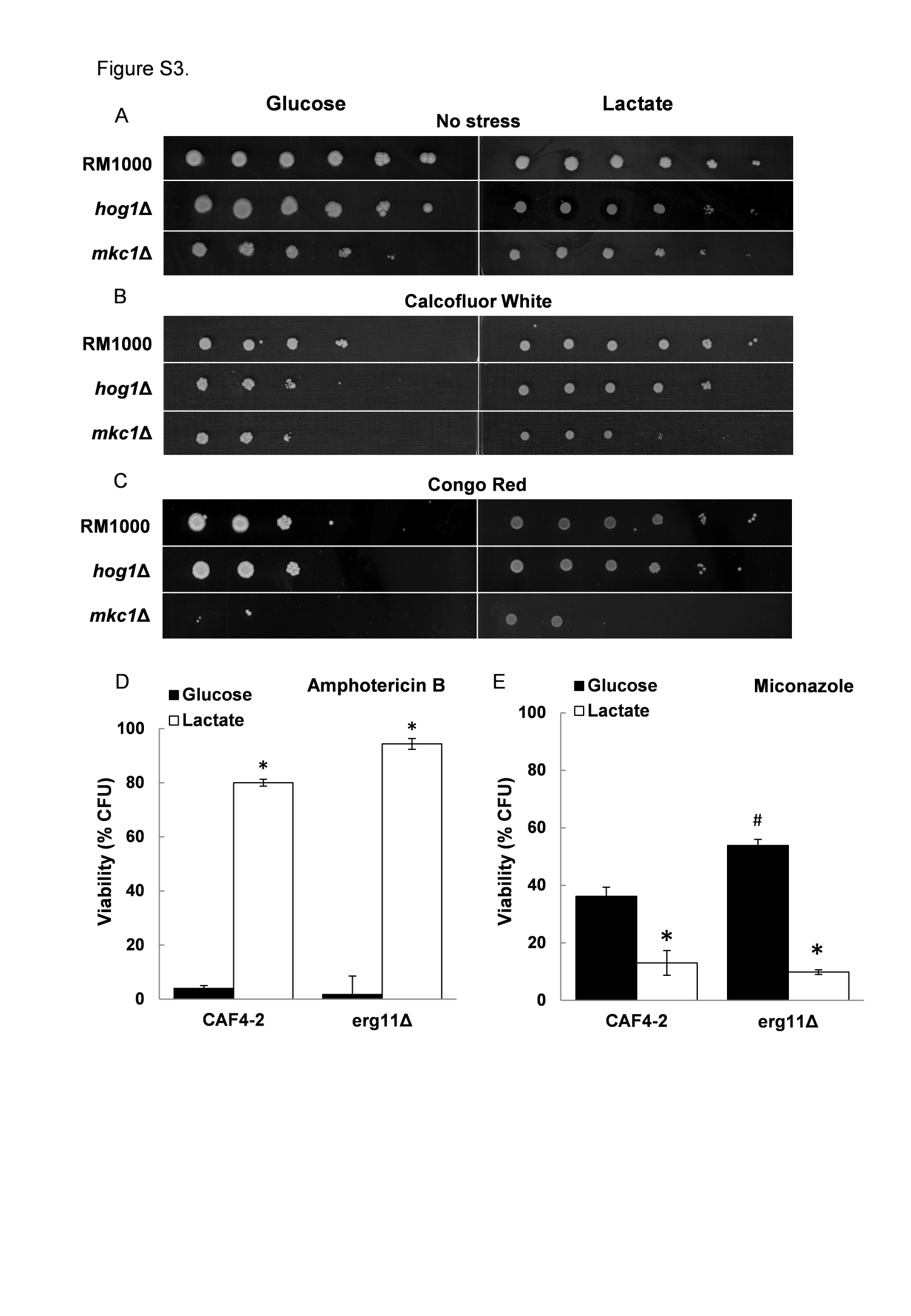


**Figure S3.** The impact of carbon source upon cell wall stress resistance is not dependent on Hog1, Mkc1 signalling or cellular ergosterol levels. Increased resistance to (B) Calcoflouor White (200 μg/ml) and (C) Congo Red (300 μg/ml) was observed in wild type (RM1000),*hog1*Δ and *mkc1*Δ cells(Table S1)grown on lactate when compared to glucose-grown cells and relative to no stress plates (A). Plates were incubated for 2-4 days at 30°C and then photographed. Pictures shown are representative of 3 independent experiments. (D) Resistance to amphotericin B (10 μg/ml) and (E) resistance to miconazole (25 μg/ml) of ergosterol depleted *erg11* cells and corresponding wild type strain.Relative to glucose-grown cells: **P*< 0.05. The resistance of the *erg11*Δ strain was compared to the corresponding wild type control,# *P*< 0.05.

**
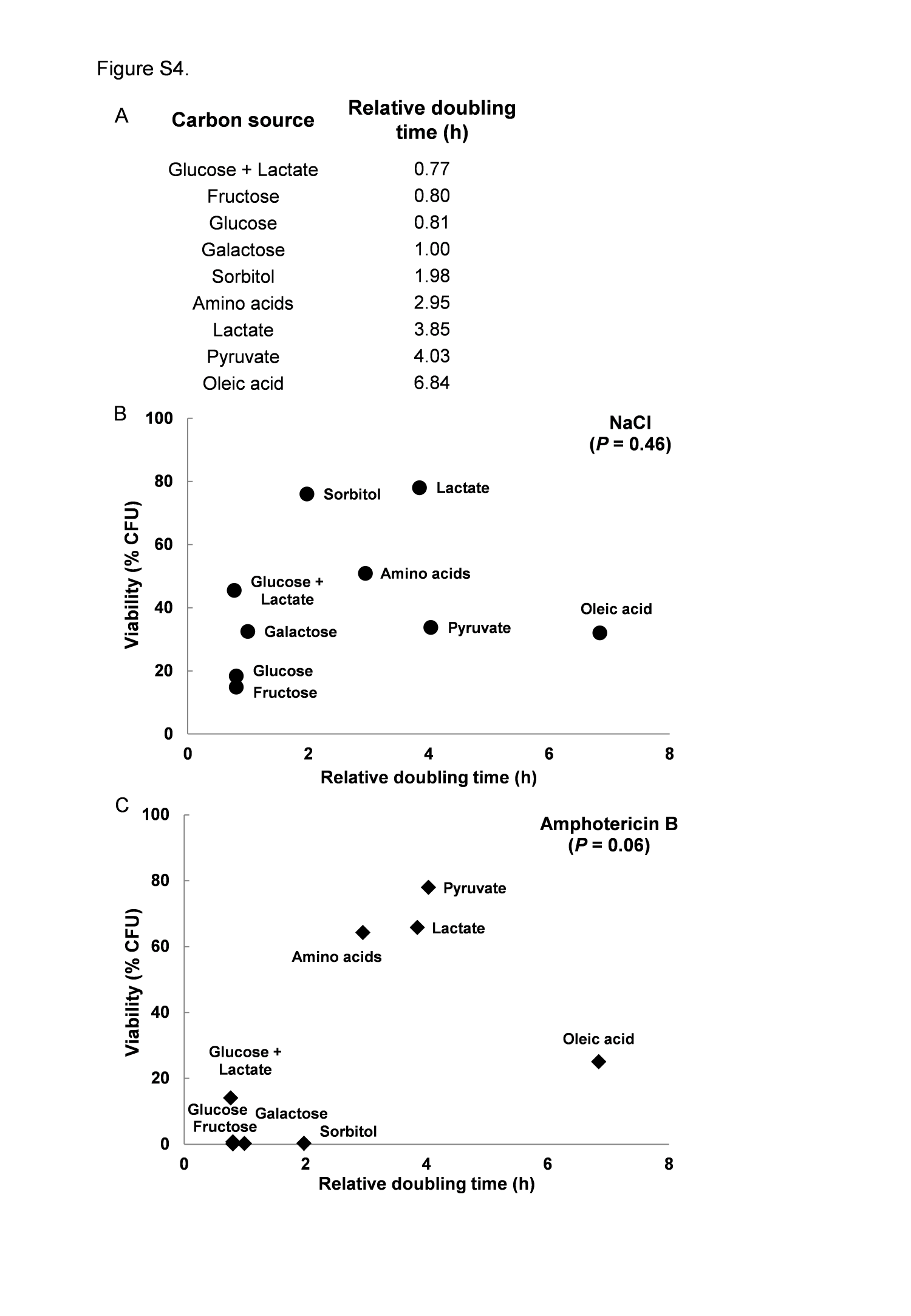

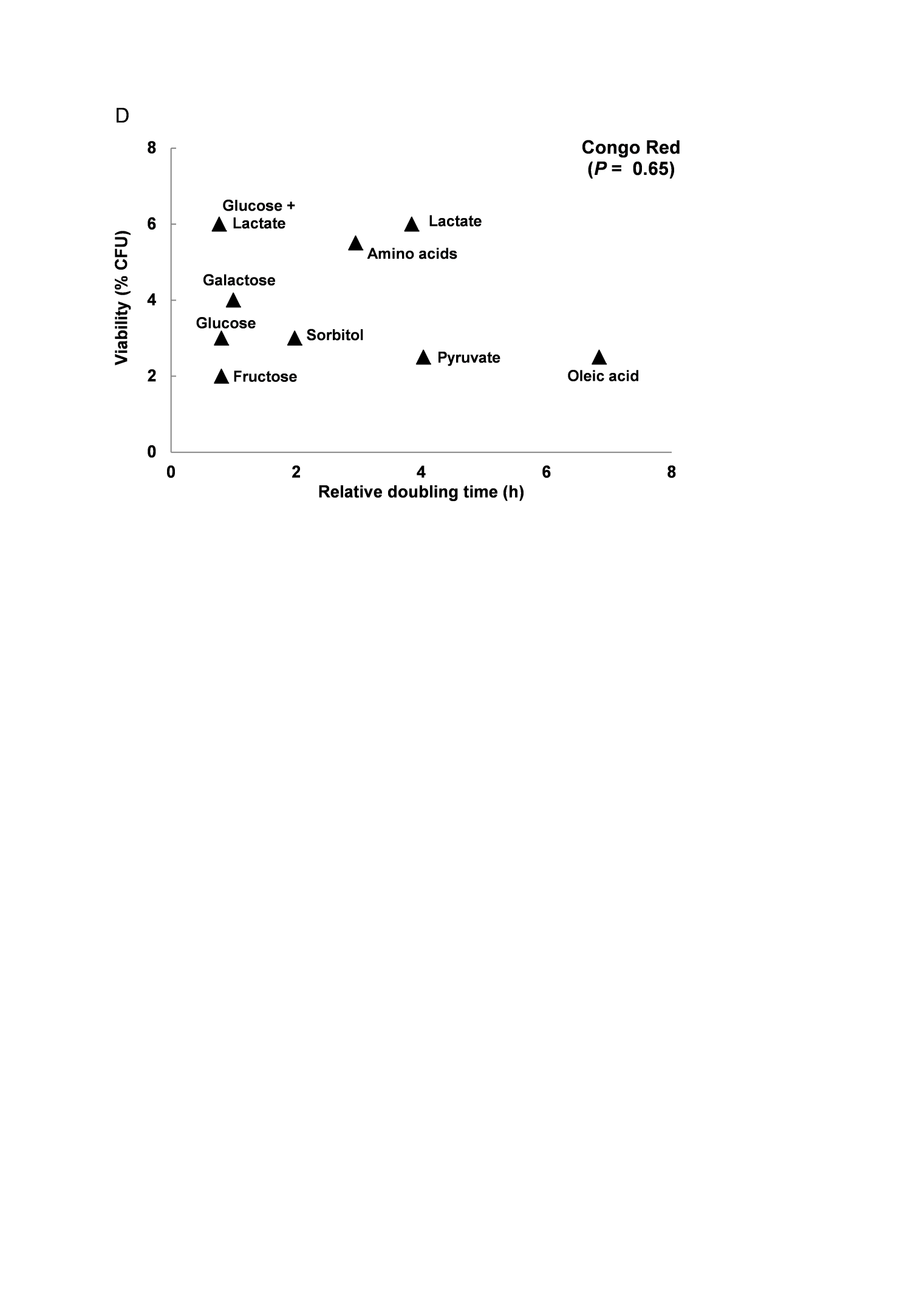
**

**Figure S4.** Stress resistance does not correlate inversely with growth rate. (A) Doubling times were calculated as the interval of time required by the cells to double from an OD_600 =_ 0.5 to 1.0. The linear equation of the exponential phase slope was used for these calculations. (B) Resistance to osmotic stress, (C) resistance to amphotericin B and (D) resistance to Congo Redof cells grown on different carbon sources were plotted against their relative doubling timesunder each growth condition. Resistance to Congo Red was assessed based on the number of dilutions (out of 6 plated) growing on Congo Red supplemented plates (Fig. 6C).No correlation was found between the rate of growth and level of resistance against the different stressors - Spearman correlation coefficient for (B) NaCl (2 M; *P* = 0.46), (C) amphotericin B (Ambisome; 10 μg/ml; *P* = 0.062) and (D) Congo Red (300 μg/ml; *P* = 0.65).
