# Supplementary material for: Host carbon sources modulate cell wall architecture, drug resistance and virulence in a fungal pathogen
Source: Cell Microbiol. 2012 Jun 5;14(9):1319–35. doi: 10.1111/j.1462-5822.2012.01813.x (PMC3465787; doi:10.1111/j.1462-5822.2012.01813.x)
Supplement: Supplementary file 2 [file cmi0014-1319-SD2.doc]

**Table S1.** Strains used in this study.

| **Species** | **Name** | **Genotype** | **Source** |
| --- | --- | --- | --- |
| *C. albicans* | RM1000 | *ura3∆::λimm434/ura3∆::λimm434, his1::hisG/his1::hisG* | Negredo *et al*., 1997 |
| *C. albicans* | RM1000 + Clp20 (JC21) | *ura3∆::λimm434/ura3∆:: λimm434, his1::hisG/his1::hisG, RSP1::Clp20 (URA3, HIS1)* | Smith et al., 2004 |
| *C. albicans* | CAF2-1 | *URA3/ura3*::imm434 *RO1/iro1*::imm434 | Sanglard *et al.*, 2003 |
| *C. albicans* | *hog1*Δ  (JC45) | *ura3∆::λimm434/ura3∆::λimm434, his1::hisG/his1::hisG, hog1::loxP-URA3-loxP, hog1::loxP-HIS1-loxP* | Smith et al., 2004 |
| *C. albicans* | *mkc1*Δ (CM1613) | *mkc1*Δ*::hisG-CaURA3-hisG/mkc1*Δ*::hisG, ura3*Δ*::λimm434/ura3*Δ*::λiimm434* | Navarro-Garcia et al., 1998 |
| *C. albicans* | *erg11 Δ* (DSY1769) | *erg11*Δ*::hisG-URA3-hisG erg11*Δ*::hisG-URA3-hisG* | Sanglard *et al.*, 2003 |
| *C. albicans* | ATCC90028 | Clinical isolate from the blood | MacCallum et al., 2009 |
| *C. albicans* | ATCC10231 | Clinical isolate from the oropharynx | Donna MacCallum |
| *C. tropicalis* | J980156 | Clinical isolate from the blood | Donna MacCallum |
| *C. lusitaniae* | SCSB5674 | Clinical isolate from the blood | Donna MacCallum |
| *C. guillermondii* | J980176 | Clinical isolate from the blood | Donna MacCallum |
| *C. glabrata* | J990679 | Clinical isolate from the blood | Donna MacCallum |
| *C. dubliniensis* | SCS59662 | Clinical isolate from the blood | Donna MacCallum |
| *C. krusei* | RB18 | Clinical isolate from the blood | Donna MacCallum |
